# Dulaglutide Alone and in Combination with Empagliflozin Attenuate Inflammatory Pathways and Microbiome Dysbiosis in a Non-Diabetic Mouse Model of NASH

**DOI:** 10.3390/biomedicines9040353

**Published:** 2021-03-30

**Authors:** Katharina Luise Hupa-Breier, Janine Dywicki, Björn Hartleben, Freya Wellhöner, Benjamin Heidrich, Richard Taubert, Young-Seon Elisabeth Mederacke, Maren Lieber, Konstantinos Iordanidis, Michael P. Manns, Heiner Wedemeyer, Matthias Hardtke-Wolenski, Elmar Jaeckel

**Affiliations:** 1 Department of Gastroenterology, Hepatology and Endocrinology, Hannover Medical School, 30625 Hannover, Germany; janine.dywicki@gmx.de (J.D.); Wellhoener.Freya@mh-hannover.de (F.W.); heidrich.benjamin@mh-hannover.de (B.H.); taubert.richard@mh-hannover.de (R.T.); Mederacke.Young-Seon@mh-hannover.de (Y.S.M.); Lieber.Maren@mh-hannover.de (M.L.); Jordanidis.Konstantinos@mh-hannover.de (K.I.); Manns.Michael@mh-hannover.de (M.P.M.); Wedemeyer.heiner@mh-hannover.de (H.W.); matthias.hardtke-wolenski@uk-essen.de (M.H.-W.); Jaeckel.Elmar@mh-hannover.de (E.J.); 2 Department of Pathology, Hannover Medical School, 30625 Hannover, Germany; hartleben.Bjoern@mh-hannover.de; 3 Department of Gastroenterology and Hepatology, Essen University Hospital, University Duisburg-Essen, 45147 Essen, Germany

**Keywords:** liver disease, non-alcoholic steatohepatitis, GLP-1 agonist, SGLT-2 inhibitor, type 2 diabetes, innate immune system, adaptive immune system, metabolic syndrome

## Abstract

Dysregulation of glucose homeostasis plays a major role in the pathogenesis of non-alcoholic steatohepatitis (NASH) as it activates proinflammatory and profibrotic processes. Beneficial effects of antiglycemic treatments such as GLP-1 agonist or SGLT-2 inhibitor on NASH in patients with diabetes have already been investigated. However, their effect on NASH in a non-diabetic setting remains unclear. With this aim, we investigated the effect of long-acting GLP1-agonist dulaglutide and SGLT-2 inhibitor empagliflozin and their combination in a non-diabetic mouse model of NASH. C57BL/6 mice received a high-fat-high-fructose (HFHC) diet with a surplus of cholesterol for 16 weeks. After 12 weeks of diet, mice were treated with either dulaglutide, empagliflozin or their combination. Dulaglutide alone and in combination with empagliflozin led to significant weight loss, improved glucose homeostasis and diminished anti-inflammatory and anti-fibrotic pathways. Combination of dulaglutide and empagliflozin further decreased MoMFLy6C^High^ and CD4^+^Foxp3^+^ T cells. No beneficial effects for treatment with empagliflozin alone could be shown. While no effect of dulaglutide or its combination with empaglifozin on hepatic steatosis was evident, these data demonstrate distinct anti-inflammatory effects of dulaglutide and their combination with empagliflozin in a non-diabetic background, which could have important implications for further treatment of NASH.

## 1. Introduction

Non-alcoholic fatty liver disease (NAFLD) is one of the most common liver diseases worldwide, which affects nearly 25% of the global adult population, and the incidence is even further increasing [1]. NAFLD includes non-alcoholic fatty liver (NAFL) as well as the more severe form, non-alcoholic steatohepatitis (NASH). A hallmark of both pathological states is the accumulation of fat in more than 5% of hepatocytes. In addition to steatosis, NASH includes also hepatocyte injury and inflammation, which can lead to end-stage liver cirrhosis and its associated complications [2]. Furthermore, NASH is a significant risk factor for the development of hepatocellular carcinoma [3]. However, a majority of the patients are dying due to cardiovascular events, which might be explained in part by the specific pathogenesis of NASH [4].

The main risk factors for NASH are central obesity, dyslipidemia, insulin resistance and type 2 diabetes mellitus (T2DM). Therefore, it was recently proposed to change the nomenclature into “metabolic associated fatty liver disease” (MAFLD) [5].

Therapeutic options in patients with NASH are still rare, but urgently needed. Currently, loss of weight and physical activity are the only established therapeutic strategies [6]. As glucose homeostasis is an important driver in the pathogenesis of NASH, antiglycemic treatments such as GLP-1 agonists have already been successfully tested for treatment of NASH in diabetic patients. GLP-1 agonists stimulate glucose-dependent insulin secretion and suppress hepatic glucagon production. They further delay gastric emptying and decrease the appetite, therefore leading to weight loss [7]. Inhibitors of the sodium-glucose cotransporter 2 (SGLT-2) such as empagliflozin are the latest novel class of anti-diabetic drugs. By inhibition of the renal SGLT-2 transporter, they prevent glucose reabsorption and therefore improve the blood glucose level and reduce glucotoxicity [8]. Some studies could already show some short-term beneficial effects of the early SGLT-2- inhibitors in patients with diabetes and NASH [9]. The combination of both GLP-1 agonist and SGLT-2-inhibitor already showed remarkable improvements of the metabolic condition in the treatment of T2DM [8] and therefore seems to be a promising therapy concept for NASH and diabetes. However, there is insufficient evidence for both anti-diabetic agents and its combination regarding the effectiveness of NASH in a non-diabetic background and their distinct pathomechanism [10]. Therefore, further studies are urgently needed. To this end, we tested the new long-acting GLP-1 agonist dulaglutide and the SGLT-2 inhibitor empagliflozin alone or in combination in a mouse model of NASH.

## 2. Materials and Methods

### 2.1. In Vivo Animal Model

Animal care and all animal experiments were executed according to protocols approved by the animal welfare commission of the Hannover Medical School, local ethics Animal Review Board (Niedersaechsisches Landesamt für Verbraucherschutz und Lebensmittelsicherheit/LAVES, Oldenburg, Germany (protocol 13/1152, 12.06.2013 and protocol 17/2456, 27.06.2017) and in accordance with the ARRIVE guidelines.

We used 6–8-week-old C57BL/6HanJ Ztm mice. Animals were fed a high-fat-high-carbohydrate (HFHC) diet with a surplus of cholesterol (Ssniff EF R/M D12330 mod.*/surwit + 1% Cholesterol; Diet#: E15771-34, S3542-E005; Ssniff, Soest, Germany) with 45 g/L 55% Fructose/45% Sucrose (Sigma, Darmstadt, Germany) in the drinking water for 16 weeks. After 12 weeks of the diet, pharmacological treatment was started with either dulaglutide (10 nmol/kg, Eli Lilly, Bad Homburg, Germany) [11], empagliflozin (10 mg/kg, Boehringer Ingelheim, Ingelheim am Rhein, Germany)) [12], a combination of dulaglutide and empagliflozin or vehicle. Further details regarding the in vivo animal model are provided in the Supplementary Data.

### 2.2. Serum Analysis

After 16 weeks of HFHC diet, an intraperitoneal glucose-tolerance test (IPGTT) was performed. Further details are provided in the Supplementary Data. For serum analysis, blood samples were collected via a retro orbital withdrawal. Aspartate aminotransferase (AST) and alanine transaminase (ALT) were determined by photometric enzyme activity assays by the Olympus AU400 Chemistry Analyzer (OLY-AU400, Hamburg, Germany) using 40–50 µL of murine serum as described before [13].

### 2.3. Liver Triglycerides

Frozen liver was minced into pieces and homogenized in standard diluent (Cayman Chemical, Ann Arbour, Michigan, USA) with 20 µg/mL Leupeptin (Serva Electrophoresis GmbH, Heidelberg, Germany) via a rod-homogenizer for 10–15 s. Homogenates were centrifuged at 4 °C for 10 min at 10,000× *g*. Supernatant was aliquoted for triglyceride and protein quantification. Colorimetric detection and quantitation of total protein was performed using the Pierce™ BCA Protein Assay Kit (Thermo Fisher Scientific Waltham, Waltham, MA, USA). Triglyceride in supernatant was detected via the Cobas^®^ 8000 modular analyzer (HITACHI/Roche, Basel, Schweiz).

### 2.4. Histology

Murine liver was fixed in formalin and embedded in paraffin. Paraffin-embedded sections (2 µm) were stained with haematoxylin and eosin (H&E) for assessment of liver histology, with periodic-acid Schiff reaction (PAS) for assessment of glycogen accumulation and with silver for evaluation of fibrosis. Afterwards, they were graded with the NAFLD-activity score (NAS). In short, sections were graded for lobular inflammation, hepatocyte ballooning and steatosis [14]. Grading was done by a blinded pathologist, who is experienced in the scoring of human NASH. Histological images were acquisited with the ZEISS Imager.M1. -microscope (ZEISS, Oberkochen, Germany), ZEISS Plan.Apochromat (20 er/08 + 10/0,45) objective (ZEISS, Oberkochen, Germany) and ZEISS Axiocam 506 mono camera (ZEISS, Oberkochen, Germany). ZEISS ZEN v2.3. blue edition (2011) software (ZEISS, Oberkochen, Germany) was used for acquisition as well as for further image processing.

### 2.5. Flow Cytometry

Livers and spleens were perfused with PBS and dissected from mice. Organs were then minced using a nylon cell strainer (70 µm, BD Falcon, Franklin Lakes, NJ, USA) and washed with phosphate-buffered saline supplemented with 2% fetal calf serum. Afterwards, spleens were lysed in red blood cell lysis buffer (Sigma-Aldrich, Munich, Germany). Intrahepatic leucocytes and monocytes were isolated by using 40/70%- percoll density gradients (Percoll, GE Healthcare, Chicago, IL, USA). Subsequently, cells were stained with fluorochrome-conjugated antibodies for multicolor fluorescence-activated cell sorting (FACS) analysis. Detailed information about the antibodies used are provided in the Supplementary Data. All acquisitions were done with a LSRII SORP interfaced to DIVA software (BD Biosciences, San Jose, CA, USA).

### 2.6. Real-Time PCR

For real-time PCR, the TaqMan Gene Expression Assay was used. Please see the Supplementary Data for a detailed list of assays. For amplification, the PreAmp master Mix (Fluidigm, PN 1005580, South San Francisco, CA, USA) was used. Amplification was performed according to the manufacturer’s guidelines. For PCR, the 48.48 Dynamic Array TM IFC, Assay loading reagent (Fluidigm, PN 85000736), GE Sample Loading Reagent (Fluidigm, PN 85000735, 85000746) and the Master Mix (TaqMan Gene Expression Master Mix, Applied Bioscience, PN 4369016, Thermofisher, Waltham, MA, USA) were used. RT-PCR was performed according to the manufacturer’s guidelines.

### 2.7. Stool Analysis

Fresh stool was collected from every animal after 16 weeks of HFHC diet. For stool collection, mice were set into a clean plastic cage and fresh stool was collected and immediately put on dry ice. Afterwards, stool was stored at −80 °C until further preparation. DNA preparation and bioinformatic analyses were performed according to the methods described before [15]. Further details are listed in the Supplementary Data [16,17,18,19].

### 2.8. Statistics

Statistical analysis was performed with GraphPad Prism v5.0. The unpaired Student 2-tailed *t*-test with Welsh-correction was applied for comparisons of differences between the means of two groups. Non-parametric Mann–Whitney test was applied for comparing the NAS-Score. The trapezoidal rule was used to determine the area under the curve (AUC). *p*-values <0.05 were considered as significant. All results are presented as mean with standard error of the mean (SEM). Graphs were created using the GraphPad Prism v5.0 program and Power Point v2010.

For analysis of the RT-PCR, the Fluidigm Real-Time PCR Analysis Software V.3.0.2 was used. Data were normalized by the mean values of the housekeeping genes glyceraldehyde-3-phosphate dehydrogenase (Gapdh) and actin β (actb) from the genes of interest. Heat map and principal component analysis (PCA) of the –delta Ct values were performed via the using Qlucore Omics Explorer v3.3 (Qlucore, Lund, Sweden). For analysis *p*-values were set to ≤0.049 for two group comparisons (*t*-test) and for multigroup comparisons (F-test) (ANOVA) (*p* < 0.05 and q < 0.2). *, difference significant with *p* < 0.05. **, difference significant with *p* < 0.01 ***, difference highly significant with *p* < 0.001. A *p*-value > 0.05 was considered to be not significant (ns).

## 3. Results

### 3.1. Dulaglutide Corrected Obesity and Reduced Adipose Tissue

**Figure 1:**
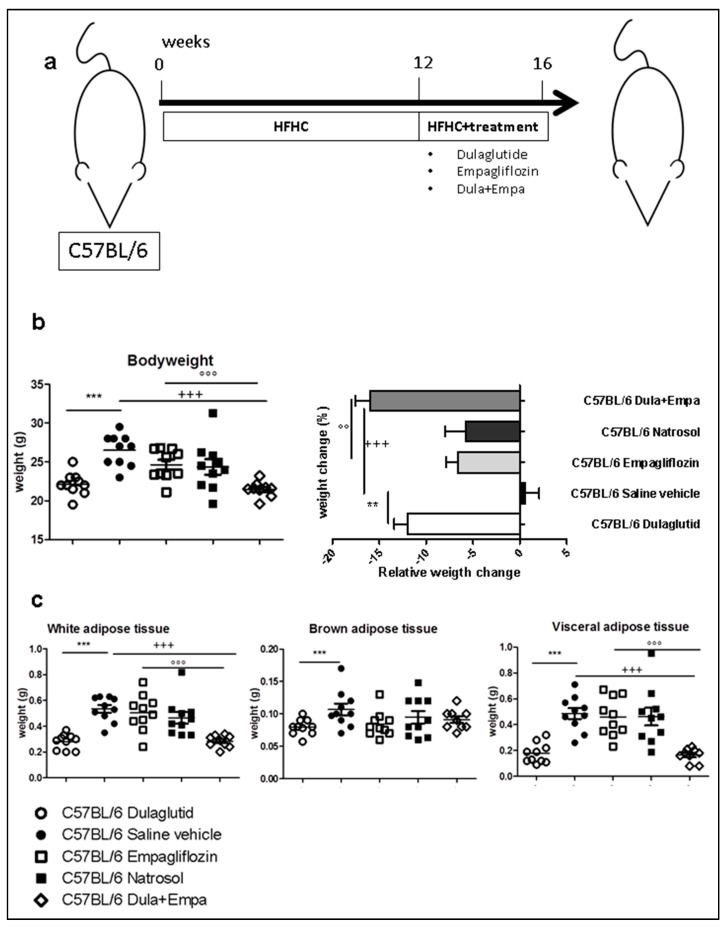
Body composition (**a**) C57BL/6 mice received a high-fat-high-fructose (HFHF) diet for a total of 16 weeks, additional pharmacological therapy administered for the last 4 weeks (week 13–16). (**b**) Body weight and relative weight change after 16 weeks of HFHC diet. (**c**) Body fat composition was analyzed by weight determination of white adipose tissue (WAT), brown adipose tissue (BAT) and visceral adipose tissue (VAT) after sacrification. Data are presented as mean ± SEM. C57BL/6 Dulaglutide: *n* = 10, C57BL/6 Saline vehicle: *n* = 10, C57BL/6 Empagliflozin: *n* = 10, C57BL/6 Natrosol: *n* = 10; C57BL/6 Dula + Empa: *n* = 9. Unpaired *t*-test with Welsh-correction was used for comparison between two groups. * *p* < 0.05; ** *p* < 0.01; *** *p* < 0.001 was used for comparison of Dulaglutide vs. Saline vehicle; + *p* < 0.05; ++ *p* < 0.01; +++ *p* < 0.001 was used for comparison of Dula + Empa vs. Saline vehicle, ° *p* < 0.05; °° *p* < 0.01; °°° *p* < 0.001 was used for the comparison of Dula + Empa vs. Empagliflozin.

Weight loss is the most important treatment option for NASH so far and therefore an important aim of potential therapeutical treatments. After 16 weeks of HFHC diet and 4 weeks of treatment (Figure 1a), we analyzed the body weight and body composition of the mice. Here, both treatment with dulaglutide as well as its combination with empagliflozin led to significant weight loss during the time of treatment and significantly reduced the total bodyweight at the end of treatment compared to saline control and treatment with empagliflozin alone (Figure 1b). In line with this finding, dulaglutide significantly reduced the amount of white adipose tissue (WAT), brown adipose tissue (BAT) and visceral adipose tissue (VAT) compared to saline control (Figure 1c). Also, the combined treatment significantly reduced WAT and VAT, but had no additional effect compared to dulaglutide (Figure 1c). Treatment with empagliflozin had no influence on the bodyweight or subset of adipose tissue. Therefore, both dulaglutide and combined treatment are effective for weight loss and reduction of adipose tissue.

### 3.2. Combination of Dulaglutide and Empagliflozin Markly Improved Glycemic Control. Dulaglutide Improved Hyperglycemia and Diminished AST-Elevation

**Figure 2:**
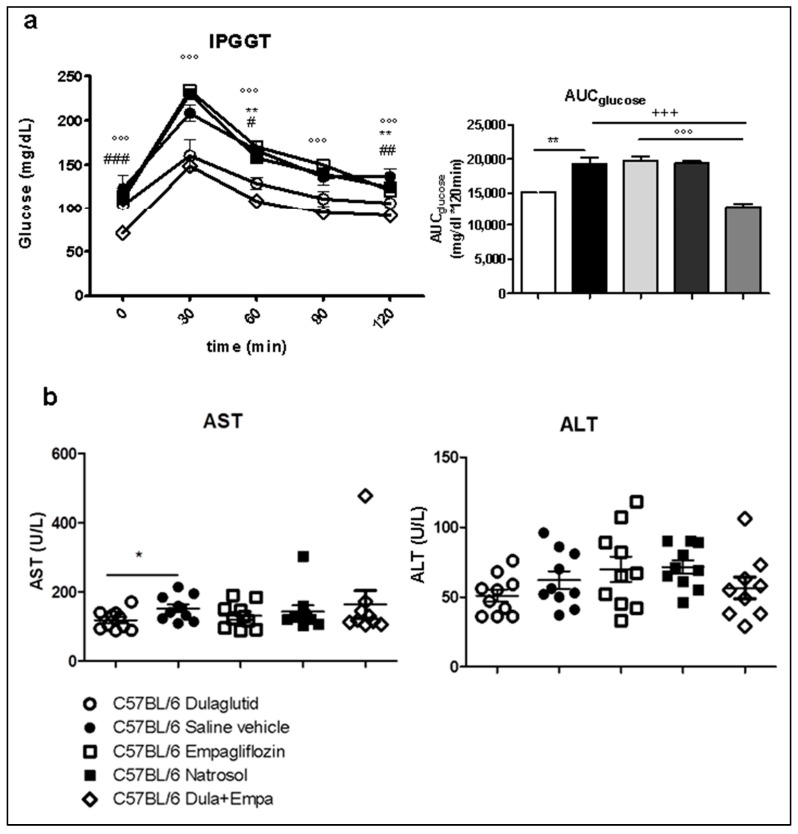
Metabolic parameters and transaminases (**a**) Intraperitoneal (i.p) glucose tolerance test. The trapezoidal rule was used to determine the area under the curve for glucose (AUC glucose). (**b**) Biochemical analyses of alanine (ALT) and aspartate (AST) transaminases in blood serum. Data are presented as mean ± SEM. Unpaired *t*-test with Welsh-correction was used for comparison between two groups. C57BL/6 Dulaglutide: *n* = 10, C57BL/6 Saline vehicle: *n* = 10, C57BL/6 Empagliflozin: *n* = 10, C57BL/6 Natrosol: *n* = 10; C57BL/6 Dula + Empa: *n* = 9; * *p* < 0.05; ** *p* < 0.01; *** *p* < 0.001 was used for comparison of Dulaglutide vs. Saline vehicle + *p* < 0.05; ++ *p* < 0.01; +++ *p* < 0.001 was used for comparison of Dula + Empa vs. Saline vehicle ° *p*< 0.05; °° *p* < 0.01; °°° *p* < 0.001 was used for the comparison of Dula + Empa vs. Empagliflozin # *p* < 0.05, ## *p* < 0.01, ### *p* < 0.001 was used for comparison of Dulaglutide vs. Dula + Empa.

Although there were no differences in the basal fasting glucose levels, dulaglutide accelerated glucose reduction after injection, so that these mice reached basal fasting glucose levels at the end of the test compared to saline vehicle-treated mice (Figure 2a). Also, the AUC glucose was significantly reduced (Figure 2b). The combined treatment of dulaglutide and empagliflozin further improved glycemic control via reduced basal fasting glucose levels as well as over the course of glucose test compared to either dulaglutide or empagliflozin alone (Figure 2a). Interestingly, empagliflozin had no effect on hyperglycemia. Therefore, these data show that dulaglutide and combination of dulaglutide and empagliflozin improve the metabolic function even in a non-diabetic setting.

We further analyzed the treatment effect on transaminases levels as a marker for hepatocyte damage. Here, treatment with dulaglutide alone significantly reduced the aspartate-aminotransferase (AST). Neither the treatment with empagliflozin nor the combined treatment influenced AST levels. No significant changes in the alanine-aminotransferase (ALT) levels were detected (Figure 2B).

### 3.3. No Histological Apparent Treatment Effects after Development of Moderate NASH

After 16 weeks of HFHC diet, the histological development of NASH was analyzed by an experienced pathologist. Paraffin-embedded liver sections were stained with haematoxylin and eosin (H&E) for assessment of liver histology, periodic-acid Schiff reaction (PAS) for assessment of glycogen accumulation and silver staining for evaluation of fibrosis (Figure S1A). All animals developed moderate steatosis, mild inflammation and mild ballooning, which defines NASH with a median NAS Score 3–4 (Figure S1B). No significant changes have been detected after any of the treatments according to the total NAS-score. All animals developed mild periportal fibrosis with a median score of 0–1 (Table S1), but no significant treatment effect was seen. Furthermore, we measured the intrahepatic triglyceride content. Surprisingly, the liver triglyceride content was not affected by any treatment (Figure S1C).

### 3.4. Combination of Dulaglutide and Empagliflozine Attenuates Activation of Innate and Adaptive Immune System

**Figure 3:**
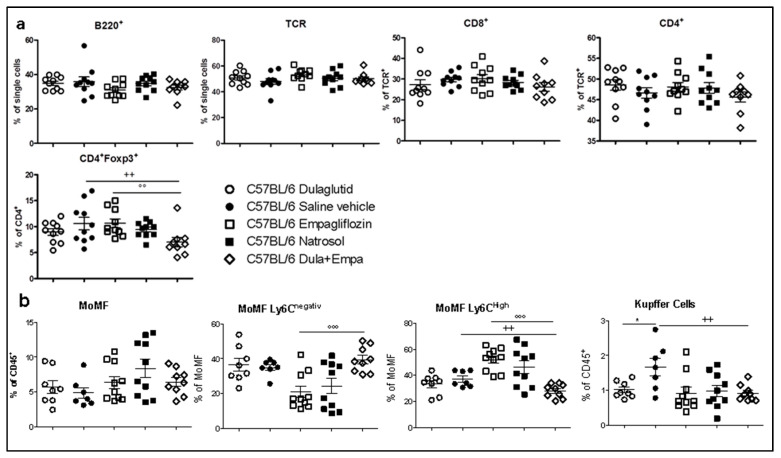
Flow cytometry analyses of the innate and adaptive immune system (**a**) Flow cytometry analyses of the adaptive immune system focusing on the intrahepatic subsets of CD4^+^ T cells and CD8^+^ T cells (**b**) Analyses of s of intrahepatic liver macrophages. Data are presented as mean ± SEM. Unpaired *t*-test with Welsh-correction was used for comparison between treatment and control group. C57BL/6 Dulaglutide: *n* = 10, C57BL/6 Saline vehicle: *n* = 10, C57BL/6 Empagliflozin: *n* = 10, C57BL/6 Natrosol: *n* = 10; C57BL/6 Dula + Empa: *n* = 9. * *p* < 0.05; ** *p* < 0.01; *** *p* < 0.001 was used for comparison of Dulaglutide vs. saline vehicle. + *p* < 0.05; ++ *p* < 0.01; +++ *p* < 0.001 was used for comparison of Dula + Empa vs. Saline vehicle. ° *p* < 0.05; °° *p* < 0.01; °°° *p* < 0.001 was used for the comparison of Dula + Empa vs. Empagliflozin.

As both the adaptive and innate immune system play an important role in the pathogenesis of NASH, we analyzed the intrahepatic subsets of immune cells using flow-cytometry. Although no significant changes regarding the adaptive immune system were seen for each single treatment (Figure 3a and Figure S2), the combined treatment decreased the number of Foxp3^+^ regulatory T (Treg) cells compared to single treatment with empagliflozin as well as control mice (Figure 3a). There was no difference in the expression of CD62L (L-Selectin) on CD4^+^ and CD8^+^ cells, which is an adhesion molecule of the selectin family, which mediates lymphocyte homing and might induce proinflammatory processes [20] (Figure S2a). Furthermore, no difference in the subsets of Ki67^+^ cells have been detected (Figure S2a).

In the subset of the cytokine production of intrahepatic lymphocytes, we could see a significant reduction in CD8^+^IL17^+^ cells as wells as a trend (*p* = 0.09) towards reduced CD4^+^IFNgamma^+^ after combined treatment compared to empagliflozin (Figure S2b). Interestingly, empagliflozin also decreased the amount of CD8^+^TNFalpha^+^. The lower Treg numbers might be further indicating lower intrahepatic IL-2 availability.

Macrophages are important drivers of chronic inflammation in the liver and contribute to the progression from simple steatosis to steatohepatitis. Polarization of monocyte-derived macrophages (MoMF) into the pro-inflammatory macrophages (MoMFLy6C^high^) is influenced by various stimuli such as fatty acids or signals from the gut–liver axis [21]. Therefore, it is important that the combined treatment with dulaglutide and empagliflozin significantly diminished the amount of proinflammatory MoMFLy6C^high^ compared to empagliflozin and control group. Conversely, the amount of MoMFLy6C^neg^ cells were significantly increased after combined treatment (Figure 3b). Kupffer cells are involved in both activation of pro-inflammatory macrophages as well as integration of signals from the gut-liver axis and lipid metabolism. Both dulaglutide as well as the combined treatment significantly reduced the amount of Kupffer cells compared to saline vehicle (Figure 3b), which is associated with diminished inflammation. In summary, combined pharmacological treatment significantly attenuated the pro-inflammatory reaction of the innate and adaptive immune system and is even more effective than any of the single treatments.

### 3.5. Dulaglutide and Combined Treatment Diminished Pro-Inflammatory Pathways and Attenuated Pro-Fibrotic Pathways, whereas Empagliflozin Enhanced Pro-Inflammatory and Pro-Fibrotic Pathways

**Figure 4:**
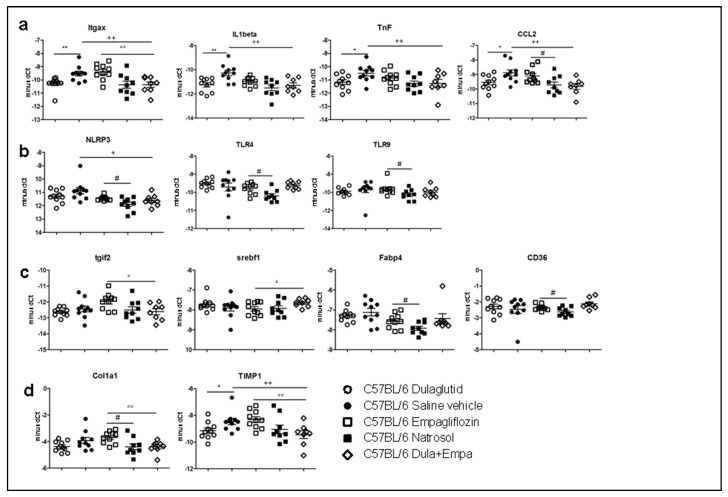
Gene expression. Normalized gene expression representing pathways of inflammation, inflammasome, fibrosis and lipid metabolism. Genes that were expressed significantly differently upon PCA were exemplarily chosen to visualize changes in the pathways of (**a**) inflammation, (**b**) inflammasome, (**c**) lipid metabolism and (**d**) fibrosis. Unpaired *t*-test with Welsh-correction was used for comparison between treatment and control group. One-way ANOVA with Tukey’s multicomparison test was used for differences between treatment groups. C57BL/6 Dulaglutide: *n* = 10, C57BL/6 Saline vehicle: *n* = 10, C57BL/6 Empagliflozin: *n* = 10, C57BL/6 Natrosol: *n* = 10; C57BL/6 Dula + Empa: *n* = 9. * *p* < 0.05; ** *p* < 0.01; *** *p* < 0.001 was used for comparison of Dulaglutide vs. Saline vehicle. # *p* < 0.05; ## *p* < 0.01; ### *p* < 0.001 was used for comparison of Empagliflozin vs. Natrosol. + *p* < 0.05; ++ *p* < 0.01; +++ *p* < 0.001 was used for comparison of Dula + Empa vs. Saline vehicle. ° *p* < 0.05; °° *p* < 0.01; °°° *p* < 0.001 was used for the comparison of Dula + Empa vs. Empagliflozin.

To determine the genetic effects of drug treatment on the pathogenesis of lipid metabolism, inflammation and development of fibrosis, we analyzed 22 genes, which represent key transcripts in the pathways of inflammation, fibrosis and fatty acid metabolism. Genes that were expressed significantly different upon PCA analysis were plotted in a heat map (Figure S3) as well as in diagrams to visualize changes in the pathways of inflammation, inflammasome, lipid metabolism and fibrosis (Figure 4a–d). Although dulaglutide did not induce histological changes, we could demonstrate anti-inflammatory and anti-fibrotic effects on a molecular level as it significantly decreased the expression of the pro-inflammatory markers CCL2, CD11c, TNF and IL-1 beta as well as the expression of the pro-fibrotic marker TIMP-1 compared to Saline vehicle (Figure 4a–d). Surprisingly, treatment with empagliflozin revealed pro-inflammatory and pro-fibrotic effects due to increased expression of the pro-inflammatory markers NLRP-3, CCL2, TLR-9 and TLR-4, CD11c, CD36 and enhancement of FABP-4 and collagen (Figure 4a–d) compared to vehicle.

Comparing the combined treatment with empagliflozin-mono, expression of pro-inflammatory markers CCL-2, CD11c, TGIF-2 as well as pro-fibrotic markers TIMP-1 and collagen was significantly decreased, suggesting a dominant effect of the dulaglutide treatment (Figure 4a–d). The expression of SREBF-1 increased after treatment with dulaglutide and empagliflozin. Although no changes between the dulaglutide group and combined treatment group could be detected, the combined treatment also significantly diminished the expression of pro-inflammatory as well as pro-fibrotic markers.

### 3.6. Microbiome Diversity Is Enhanced by Dulaglutide and Combination of Dulaglutide and Empagliflozine

Besides lipid and glucose metabolism, the gut–liver axis also contributes to the progression of NASH. In particular, microbiome dysbiosis causes flux of microbial endotoxins to the liver, which then further promotes pro-inflammatory and pro-fibrotic processes [22]. Therefore, we analyzed the changes in the microbiome after treatment. In total, we analyzed 18,656 reads per sample and could classify six different phyla and 47 different genera. Only 0.02% of reads could not be assigned at phylum level.

**Figure 5:**
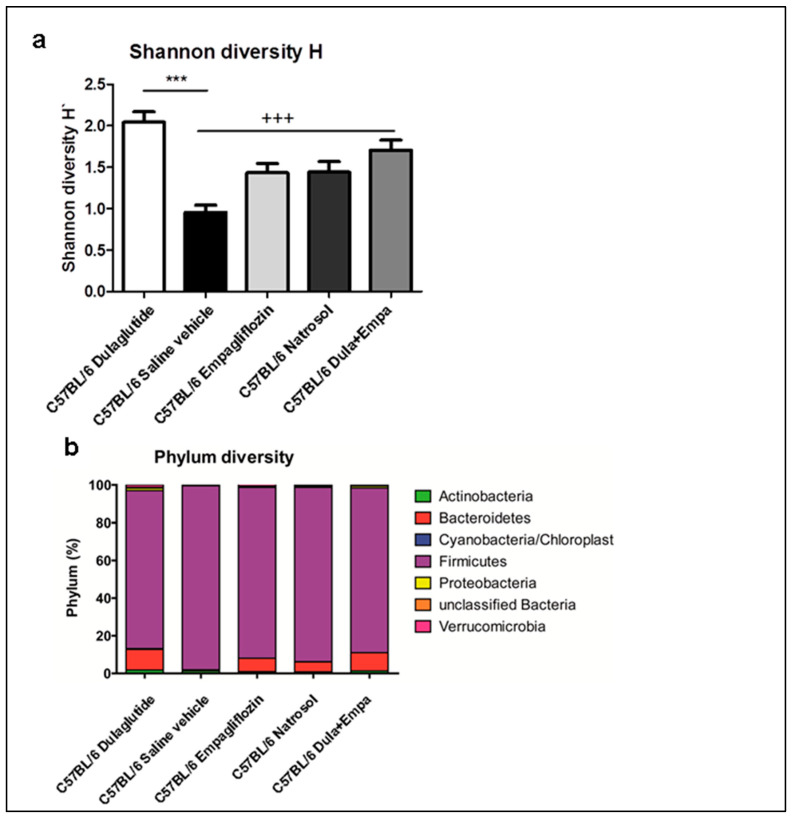
Microbiome diversity (**a**) Analysis of the genus diversity of intestinal microbiota using the Shannon H score. Data are presented as mean ± SEM. Unpaired *t*-test with Welsh-correction was used for comparison between treatment and control group. (**b**) Detailed composition of the phylum diversity. C57BL/6 Dulaglutide: *n* = 10, C57BL/6 Saline vehicle: *n* = 10, C57BL/6 Empagliflozin: *n* = 10, C57BL/6 Natrosol: *n* = 10; C57BL/6 Dula + Empa: *n* = 9. * *p* < 0.05; ** *p* < 0.01; *** *p* < 0.001 was used for comparison of Dulaglutide vs. Saline vehicle. + *p* < 0.05; ++ *p* < 0.01; +++ *p* < 0.001 was used for comparison of Dula + Empa vs. Saline vehicle.

Alpha diversity on genus level was significantly augmented after dulaglutide treatment compared to the saline control group in both Shannon- and Simpson-diversity index (Figure 5a). Also, combined treatment enhanced microbiome dysbiosis compared to saline vehicle. On phylum level, significant differences in beta diversity could be observed when comparing dulaglutide treatment with the saline vehicle (Figure 5b). Interestingly, an increase of *Bacteriodetes* and a concomitant lower abundance of *Firmicutis* were seen, which are both related to lower insulin resistance and obesity in humans and mice. Additionally, the increase of *Bacteriodetes* and concomitant reduction of *Firmicutis* was also seen after empagliflozin treatment and combined treatment.

Analyses of microbiome similarities were performed on genus level. Here, we could show significant differences between the dulaglutide-group and the control group. Determination of the relative abundance of different genera, treatment with dulaglutide as well as treatment with empagliflozin and combined treatment were associated with significant differences in the relative abundance of different genera (Table S2). However, there are more differences between combined treatments with empagliflozin than with dulaglutide, which underlines the dominant effect of dulaglutide.

To summarize, we could show significant improvement of microbiome dysbiosis after dulaglutide treatment and its combination with empagliflozin, which correlates with diminished activation of pro-inflammatory and pro-fibrotic pathways in our previous analyses.

## 4. Discussion

To our knowledge, this is the first study investigating the effects of the new, long-acting GLP-1 agonist dulaglutide and the SGLT-2 inhibitor empagliflozin alone and in combination in a dietary mice model for non-alcoholic steatohepatitis.

Dulaglutide corrects obesity and improves glycemic control even in a non-diabetic mouse model and therefore improves important risk factors for the pathogenesis of NASH. The combination therapy with GLP-1 agonist and SGLT-2 inhibitor is meant to be a promising treatment as their effects are supplementary.

A few studies have already examined this combination and have shown beneficial effects on glycemic control and body weight in humans [23]. In our study, we could confirm beneficial effects of combined treatment on glycemic control compared to monotherapy with GLP-1. However, the effect of the combination on the HbA1c- reduction in the mentioned study was less than the additive effect of SGLT-2 inhibition alone plus GLP-1- agonist alone.

Furthermore, our data clearly demonstrated anti-inflammatory effects of both dulaglutide and the combination of dulaglutide and empagliflozin. A previous study also demonstrated strong anti-inflammatory effect of GLP-1 agonist as an important pathway to improve NASH. Furthermore, they postulated that this effect might be partly independent from body weight reduction and glycemic improvement [24].

In our study, the anti-inflammatory effects are mediated by various pathways. Kupffer cells, the resident macrophages of the liver, play a critical role in the onset of NASH, as their activation results in the release of various inflammatory mediators, such as chemokine (C-C motif) ligand (CCL2) and TNF alpha [25]. CCL-2 mediates the recruitment of MoMF. These MoMFs can be further divided into proinflammatory monocytes (MoMF Ly6C^high^) and anti-inflammatory monocytes (MoMF Ly6C^low^). In NASH, proinflammatory MoMF strongly promote chronic inflammation and therefore play a critical role in pathogenesis of NASH. In line with the decrease of Kupffer cells, we observed a decrease of CCL2-gene expression in the liver as a marker for decreased inflammation. In addition, the expressions of TNF alpha and IL1beta were significantly decreased after dulaglutide treatment. Previously it was shown that GLP-1 agonist liraglutide modulated the Kupffer cell M2-polarization and inhibits inflammasome activation, which might contribute to the anti-inflammatory effect of dulaglutide [26]. Additionally, another study investigating liraglutide in NASH showed improvement of ER stress, which might also contribute to the anti-inflammatory action of GLP-1 agonists [27].

Importantly, we could show an even stronger attenuation of pro-inflammatory activation of innate and adaptive immune system marked by decreased Treg population as well as decreased pro-inflammatory macrophages (MoMF Ly6C^high^) and Kupffer cells in a combined treatment group. Although our data did not show any significant effect of empagliflozin on innate immunity, a previous study in a diabetic mouse model showed improvement of inflammation and insulin resistance by reduction of pro-inflammatory macrophages [28]. Therefore, the combination of dulaglutide and empagliflozin has additive effects on pro-inflammatory process.

Previous animal and human studies already suggested that the gut microbiome plays an important role in the pathogenesis of NASH as the flux of microbial endotoxins to the liver further promotes pro-inflammatory and pro-fibrotic processes [29]. Most importantly, reduced microbial diversity and an increase of bacteria belonging to the *Firmicutes* phylum as well as decreased of *Bacteriodetes* seem to be associated with NASH [30]. Therefore, the increased *Firmicutes:Bacteriodetes* ratio, which is mostly pronounced in the saline vehicle group of our study, might be associated with NAFLD development. In the current study, *Firmicutes* are the very dominant phylum and are even more dominant compared to other studies [30], with a very low overall abundance of *Bacteriodetes*. In particular, lack of *Bacteriodetes* in the saline group is surprising. As other studies demonstrated microbiome dysbiosis after high-salt diet [31], the application of saline might also influence the microbiome and explain this finding. Most important, we could show a significant improvement of microbiome diversity after dulaglutide treatment and in particular with an increased relative abundance of *Bacteriodetes,* but also after combined treatment. Previous studies showed a correlation between weight loss and increase of abundance of *Bacteriodetes* as well as an association between improvement of metabolic situation and increase of *Bacteriodetes* after GLP-1 treatment [32]. Furthermore, the significant increase of *Akkermansia spp*. after dulaglutide treatment might be in correlation to improvement of the metabolic situation as *Akkermansia spp*., which is a mucin-degrading short fatty acid-producing species, is decreased in obesity and showed negative correlation to markers of gut permeability and inflammation [29]. However, the gut microbiome in mice might also be influenced by different environmental factors such as diet [33], which should be considered in terms of interpretation. In summary, the improvement of microbiome dysbiosis after dulaglutide and combined treatment consorts the improvement of metabolic dysfunction and strengthens their anti-inflammatory effects. Furthermore, the combination showed even stronger anti-fibrotic effects by downregulation of Col1a1 and TIMP-1. Although we could demonstrate significant attenuation of anti-inflammatory and anti-fibrotic pathways, no improvements on the intrahepatic steatosis and the histological outcome of NASH were detected for any of the treatments. Regarding the intrahepatic steatosis, previous data suggest that GLP-1 agonists improve hepatic steatosis by reduction of de novo lipogenesis and improving insulin signaling pathways [34]. However, we could not show any significant effect of dulaglutide on hepatic steatosis nor in pathways of lipogenesis. In line with our findings, quantitative hepatic steatosis in mice model of NASH using MCD diet was also unaffected by GLP-1 treatment [35], although GLP-1 treatment attenuated hepatic inflammation. However, it has to be mentioned that MCD diet induces NASH by different pathways compared to HFHC diet used in our model [36] and might not be suitable to represent the common features of the metabolic syndrome. On the other hand, another study investigating GLP-1 agonist in diabetic mice models showed significant improvement of intrahepatic steatosis, but only mild histological improvement of inflammation [37]. Furthermore, improvement of hepatic steatosis seems to be associated with the amount of weight loss.

As insulin resistance promotes dysregulation of peripheral lipolysis and de novo lipogenesis, which then leads to accumulation of free fatty acids in the liver [2], the effects of GLP-1 agonists on hepatic steatosis might be less prominent when hyperinsulinemia and hyperglycemia are not present and seem not to be directly correlated with their anti-inflammatory properties. Furthermore, the degree of effectiveness of GLP-1 agonists seems to be according to the manifestation of diabetes and obesity, which is important to take into account when comparing different mouse models.

A shortcoming of the study might be the moderate histological manifestation of NASH in this mice model. It is well known from other forms of hepatitis, e.g., autoimmune hepatitis, that histological changes follow biochemical improvements, but months later. Therefore, a prolonged duration of diet as well as a prolonged duration of treatment might be required to determine the histological effects.

The pathogenesis of NASH is a complex interaction between environmental factors and genetic determinants. On the one hand, this complex interplay could not be completely represented by a single animal model [36]. However, on the other hand animal models are extremely helpful to analyze distinct pathways without interference as such analyses are not possible in the human setting. Therefore, our animal model could reflect distinct pathways in the pathogenesis of NASH in a non-diabetic setting.

Beyond weight loss and improvement of insulin resistance, the positive effects of GLP-1 agonists also seem to be multifactorial-mediated with additional pleiotropic effects, especially on improvement of cardiovascular disease. As our mouse model replicates only moderate features of the metabolic syndrome in a non-diabetic setting, some pleotropic effects of GLP-1 agonist might not be present in our model.

Previous meta-analysis of human studies investigating the impact of GLP-1 agonist in NASH showed promising results with improvement of hepatic steatosis, histological resolution of NASH and decrease of transaminases [38,39]. However, it should be mentioned that the majority of the patients were diabetic.

Previous studies with a lesser amount of diabetic patients [40,41] could show improvement of metabolic parameters and resolution of NASH, but could not show histological improvement of fibrosis, which is in line with our findings. Therefore, the beneficial effects of GLP-1 agonist might be more pronounced in patients with diabetes. Furthermore, as GLP-1 agonists mainly improve the underlying harmful metabolic dysfunction first, the entire long-term improvement of NASH and fibrosis might require even more prolonged treatment and follow-up period.

We could not detect any effect of empagliflozin treatment, neither on the metabolic situation nor on the histological outcome of NASH. Instead, empagliflozin significantly increased expression of pro-inflammatory and pro-fibrotic genes. These data are partly unexpected, as previous data from human and animal studies with SGLT-2 inhibitors showed improvement of NASH [42]. To rule out technical issues, the pharmacological effect of empagliflozin was proven by increased glucosuria after empagliflozin treatment (Figure S4). The absent effect on body weight can partly be explained by the mild total weight gain in both oral gavage treatment groups, so that the daily oral gavage might also decrease the overall food intake. So far, empagliflozin has been only tested for NASH in a streptozotocin-induced NASH-mouse model [12], where it showed improvement of hepatic steatosis and fibrosis. Compared to our mouse model, the streptozotocin-induced mouse model provides a severe diabetic phenotype, but without any other features of the metabolic syndrome. A recently published study in patients without diabetes also revealed no significant reduction of hepatic steatosis after empagliflozin treatment compared to the control group [43]. In contrast to other anti-diabetic agents, SGLT- inhibitors are independent of beta-cell function as they inhibit the sodium-glucose cotransporter 2 (SGLT- 2) in the proximal tubulus of the kidney [8,44]. In human studies, the glucose-lowering effect of empagliflozin is more potent in patients with higher HbA1c-values [45]. In line with our findings, recent clinical trials of SGLT-2 inhibitors in non-diabetic patients with chronic kidney disease or heart failure have also demonstrated divergent results regarding the metabolic effects in non-diabetic patients [46,47] meaning that the metabolic effects in non-diabetic patients are not as strong as in diabetic patients [48], although they had similar preserved cardiorenal protection. Overall, the effect and indication of SGLT-2-inhibitors for non-diabetic patients in regard to NASH should be critically reviewed and therefore, further studies are needed. So far, the combination of GLP-1 agonist and SGLT-2- inhibitors has been successfully tested in patient with type 2 diabetes [49,50,51], where combination therapy further improved hyperglycemia and obesity. This is mainly explained by their synergistic mechanism of action. While GLP-1 agonists stimulate insulin secretion and suppress glucagon production, SGLT-2-inhibitors improve hyperglycemia by enhanced glucosuria [8].

Nevertheless, the additive effect was less pronounced in using lower dosing and more pronounced in severe diabetic situations in these studies. Therefore, the lack of treatment effects by empagliflozin might explain the limited effect of combined treatment compared to dulaglutide-mono treatment with only slightly additional effects on the one hand. On the other hand, one study could show that the SGLT-2-inhibitors induced increase of endogenous glucose production could not be suppressed by combination with GLP-1 agonist [52], which might attenuate the additional benefits, in particular in a non-diabetic situation.

Furthermore, in the studies mentioned above, no additional effects on lipid level were detected. As dyslipidemia is an important risk factor for development of steatohepatitis, in particular in the absence of diabetes, this might also contribute to the marginal benefit on NASH.

Further limitation of the study might be the lack of an equal control group for the combined treatment. However, comparing both control groups for the individual treatments, we could not see any significant differences. Therefore, we might expect a similar outcome after a combined treatment with saline and Natrosol compared to single treatment.

In conclusion, our results did demonstrate no beneficial effects of empagliflozin on the development of NASH in a non-diabetic setting. Furthermore, these data suggest that GLP-1 agonists and SGLT-2 inhibitors are less effective for the prevention of hepatic steatosis in a non-diabetic background compared to previous studies. Nevertheless, the study demonstrates important anti-inflammatory effects of dulaglutide and the combination of dulaglutide and empagliflozine by modulating pro-inflammatory immune response and microbiome dysbiosis. As development of hepatic fibrosis is strongly triggered by inflammation, this might have important implications for the treatment of NASH, e.g., in combination with anti-steatotic treatment. Therefore, further studies are needed to investigate the long-term impact of both agents on NASH and hepatic fibrosis in a non-diabetic background.

## Data Availability

The data presented in this study are available on request from the corresponding author.

## Supplementary Materials

The following are available online at https://www.mdpi.com/article/10.3390/biomedicines9040353/s1; Supplementary Materials and Methods; Figure S1: Histological outcome of NAFLD after HFHC diet; Figure S2: Innate and adaptive immune system; Figure S3: Heat map.; Figure S4: Glucosuria after empagliflozin treatment; Table S1: Histological Scoring for NASH; Table S2: Analyzing the relative abundance of different genera.
