# Supplementary material for: Dulaglutide Alone and in Combination with Empagliflozin Attenuate Inflammatory Pathways and Microbiome Dysbiosis in a Non-Diabetic Mouse Model of NASH
Source: Biomedicines. 2021 Mar 30;9(4):353. doi: 10.3390/biomedicines9040353 (PMC8066839; doi:10.3390/biomedicines9040353)
Supplement: Supplementary file 1 [file biomedicines-09-00353-s001.pdf]

Dulaglutide alone and in combination with empagliflozin attenuate inflammatory pathways and microbiome dysbiosis in a non-diabetic mouse model of NASH

Katharina Luise Hupa-Breier[1], Janine Dywicki[1], Björn Hartleben[3], Benjamin Heidrich[1], Freya Wellhöner[1], Richard Taubert[1], Young-Seon Elisabeth Mederacke[1], Maren Lieber[1], Konstantinos Iordanidis[1], Michael P. Manns[1], Heiner Wedemeyer[1], Matthias Hardtke-Wolenski[1,2,], Elmar Jaeckel[1]

Table of content

Supplementary material and methods 2
- Animals 2
- Intraperitoneal glucose-tolerance test 3
- Flow cytometry analyses 3
- Nuclein acid isolation 4
- TaqMan® Assays 4
- Stool analysis 5

Supplementary figures and tables 7
- Supplementary Figure S1 7
- Supplementary Figure S2 9
- Supplementary Figure S3 10
- Supplementary Figure S4 10
- Supplementary Table S1 11
- Supplementary Table S2 12

Supplementary material and methods

Animals

All animal experiments were executed according to protocols approved by the animal welfare commission of the Hannover Medical School and local ethics Animal Review Board (Niedersaechsisches Landesamt für Verbraucherschutz und Lebensmittelsicherheit/LAVES, Oldenburg,Germany) coverd by study number 13/1152 and 17/2456.

We used 6-8 week old female C57BL/6HanJ Ztm mice, which were bred and housed under specific pathogen-free conditions with controlled temperature and dark-light cycle at the Central Animal Facility (Ztm) of the Hannover Medical School.

Animals were fed *ad libidium* with a high fat-high carbohydrate (HFHC) diet with a surplus of cholesterol (Ssniff EF R/M D12330 mod.*/surwit + 1% Cholesterol; Diet#: E15771-34, S3542-E005, Ssniff, Soest, Germany) with 45g/L 55% Fructose/45% Sucrose (Sigma, Darmstadt, Germany in the drinking water *ad libidum* for 16 weeks. After 12 weeks of the diet, mice were randomly divided into 5 treatment groups for the last 4 weeks. All animals were included into the study. Sample size was calculated by biostatistical analyses based on previous experiments. Mice were either treated with dulaglutide (10nmol/kg/3xper week; Eli Lilly, Bad Homburg, Germany) (n=10) in saline intraperitoneally (i.p.) or treated with saline i.p. as control group in the same volume (n=10). Another treatment group (n=10) was treated daily with empagliflozin (10mg/kg; Boehringer Ingelheim, Ingelheim am Rhein, Germany) in 0,5% Hydroxyethylcellulose (Natrosol, Caelo, Hilden, Germany) administered by oral gavage. The control group (n=10) was treated with 0,5% Hydroxyethylcellulose by oral gavage with the same volume. Intragastric gavage administration was carried out with conscious animals, using straight gavage needles appropriate for the animal size. The biological effect of empagliflozin was proven by measuring the urine glucose content (Combur-Test, Roche, Basel, Schweiz). Another group (n=9) was treated both with daily empagliflozin p.o.(10mg/kg; Boehringer Ingelheim, Ingelheim am Rhein, Germany) and with dulaglutide (10nmol/kg/3xper week; Eli Lilly, Bad Homburg, Germany). The doses in dulaglutide and empagliflozine were

selected according to previous studies [1, 2]. All mice were sacrificed after 16 weeks of diet by CO_2 inhalation followed by cervical dislocation to minimize animal discomfort. Blood was collected via puncture of the retrobulbar plexus just after death. Liver, spleen and ectopic adipose tissue (brown adipose tissue-BAT, white adipose tissue-WAT, visceral adipose tissue-VAT) were collected for further investigations. Mice were weighed non-fasted.

Intraperitoneal glucose-tolerance test

After 16 weeks of HFHC diet, an intraperitoneal glucose-tolerance test (IPGTT) was performed. After 12 hours of fasting, fasting blood glucose levels were measured using blood from the tail vein (One Touch Ultra, LifeScan, Milpitas, CA, USA). Following this, 1mg glucose/g bodyweight was administered intraperitoneally (i.p.). Blood glucose levels were measured at 30, 60, 90 and 120 minutes after injection, using a blood glucose test strip (One Touch Ultra/LifeScan, Milpitas, CA; USA)).

Flow cytometry analyses

**Antibody**	**Supplier**	**Cat. Number**	**Clone**
CD3	BioLegend, San Diego, CA, USA	100306	145-2C11
CD4	BioLegend, San Diego, CA, USA	GK1.5	GK1.5
CD8	BioLegend, San Diego, CA, USA	100414	HIT8a
B220	BioLegend, San Diego, CA, USA	553091	RA3-6B2
CD62L	BioLegend, San Diego, CA, USA	104441	MEL-14
INF gamma	BioLegend, San Diego, CA, USA	505810	XM61.2
IL4	BioLegend, San Diego, CA, USA	504104	11B11
IL17	BioLegend, San Diego, CA, USA	506916	TCH-18H10.1
Ly6G	BioLegend, San Diego, CA, USA	127607	1A8
CD11b	BioLegend, San Diego, CA, USA	101223	M1/70
F4/80	BioLegend, San Diego, CA, USA	123116	BM8
Ly6C	BioLegend, San Diego, CA, USA	128017	HK1.4
CD115	BioLegend, San Diego, CA, USA	135512	AFS 98

CD11c	BioLegend, San Diego, CA, USA	117333	N418
CD45	BioLegend, San Diego, CA, USA	559864	30-F11
NK1.1	BDBioscience , , San Jose, California, USA	553165	PK136
Ki67	BDBioscience , , San Jose, California, USA	558615	BS6
TNF alpha	BDBioscience , , San Jose, California, USA	554419	
CD19	BDBioscience, , San Jose, California, USA	115507	6D5
Foxp3: FJK-16s	eBioscience; ThermoFisherScientific, Waltham, Massachusetts, USA	12-5773-82	FJK-16s

Nuclein acid isolation

Total nuclein acid isolation from 30mg of frozen liver tissue was performed using the RNeasy Mini Kit (#74106 Qiagen, Hilden, Germany).

TaqMan® Assays

All TaqMan® Assays were purchased from Thermo Fisher Scientific (Waltham, Massachusetts, USA Assays are tagged with FAM and MGB and have murine specificity.

gene symbol	Assay ID	Gene name
Nfe2l2	Mm00477784_m1	Nuclear factor (erythroid-derived 2)-like 2
CD36	Mm00432403_m1	CD36
Tlr4	Mm00445273_m1	Toll-like receptor 4
Tnf	Mm00443258_m1	Tumor necrosis factor alpha
Srebf1	Mm00550338_m1	Sterol regulatory element binding transcription factor 1
Tlr9	Mm00446193_m1	Toll-like receptor 9
Il1b	Mm00434228_m1	Interleukin 1 beta
Nlrp3	Mm00840904_m1	NLR family, pyrin domain containing 3
Fabp4	Mm00445878_m1	Fatty acid binding protein 4, adipocyte
Fgf21	Mm00340165_g1	Fibroblast growth factor 21
Timp1	Mm01341361_m1	Tissue inhibitor of metalloproteinase 1
Ccl2	Mm00441242_m1	Chemokine (C-C motif) ligand 2
TbX21	Mm00450960_m1	T-box 21
Foxp3	Mm00475162_m1	Fork head box P3
Il17a	Mm00439618_m1	Interleukin 17A
Col1a1	Mm00801666_g1	Collagen, type I, alpha 1
Itgax	Mm00498698_m1	Integrin alpha X
Mmp2	Mm00439498_m1	Matrix metallopeptidase 2

Tgfb2	Mm00436955_m1	Transforming growth factor, beta 2
Acta2	Mm00725412_s1	Actin, alpha 2, smooth muscle, aorta
Il23	Mm01160011_g1	Interleukin 23
Gapdh	Mm99999915_g1	Glyceraldehyde-3-phosphate dehydrogenase
Actb	Mm00607939_s1	Actin, beta
Gata3	Mm00484683_m1	GATA binding protein 3

Stool analysis

DNA extraction was performed using the TRIzol Reagent (Ambion, Carlsbad, CA, USA)) according to the manufacturer's guidelines. Additionally, mechanical lysis using Lysing Matrix E (MP 10 Biomedicals, Eschwege, Germany) in a Fast Prep-24 instrument (MP Biomedicals, Eschwege, Germany) for 60 s at an intensity 11 setting of 5.5 M/S [3] was added. Previously described primers for the V1 and V2 region of the 12 16S rRNA gene were used for amplification [4]

For the bioinformatics analysis, sequencing was performed using the Illumina MiSeq platform and bioinformatic processing of paired end reads were done. Raw reads were merged and subsequently aligned using MOTHUR (gotoh algorithm using the SILVA reference database) prior to pre-clustering (diffs=2) [5]. Phylotypes exhibiting an average abundance of ≥ 0.001% and a sequence length ≥ 250bp were considered for follow up analysis. Phylotypes were classified using RDP's naive Bayesian classifier applying a confidence threshold of 80% [6]. Sequences from phylotypes that were not annotated down to the genus level were subjected to RDP's Seqmatch allowing for some additional manual annotations at this level as previously described [4]. Sequence counts were merged at taxonomic levels within each sample. Count tables were subsampled to lowest number of sequences using the function *rarefy_even_depth (rngseed=TRUE)* from R's package *phyloseq.*

1. Jojima, T., et al., *Empagliflozin (an SGLT2 inhibitor), alone or in combination with linagliptin (a DPP-4 inhibitor), prevents steatohepatitis in a novel mouse model of non-alcoholic steatohepatitis and diabetes.* Diabetol Metab Syndr, 2016. **8**: p. 45.
2. Byrd, R.A., et al., *Chronic Toxicity and Carcinogenicity Studies of the Long-Acting GLP-1 Receptor Agonist Dulaglutide in Rodents.* Endocrinology, 2015. **156**(7): p. 2417-28.

3. Chomczynski, P., *A reagent for the single-step simultaneous isolation of RNA, DNA and proteins from cell and tissue samples.* Biotechniques, 1993. **15**(3): p. 532-4, 536-7.
4. Camarinha-Silva, A., et al., *Comparing the anterior nare bacterial community of two discrete human populations using Illumina amplicon sequencing.* Environ Microbiol, 2014. **16**(9): p. 2939-52.
5. Cole, J.R., et al., *Ribosomal Database Project: data and tools for high throughput rRNA analysis.* Nucleic Acids Res, 2014. **42**(Database issue): p. D633-42.
6. Wang, Q., et al., *Naive Bayesian classifier for rapid assignment of rRNA sequences into the new bacterial taxonomy.* Appl Environ Microbiol, 2007. **73**(16): p. 5261-7.

Supplementary figures and tables

Supplementary Figure S1

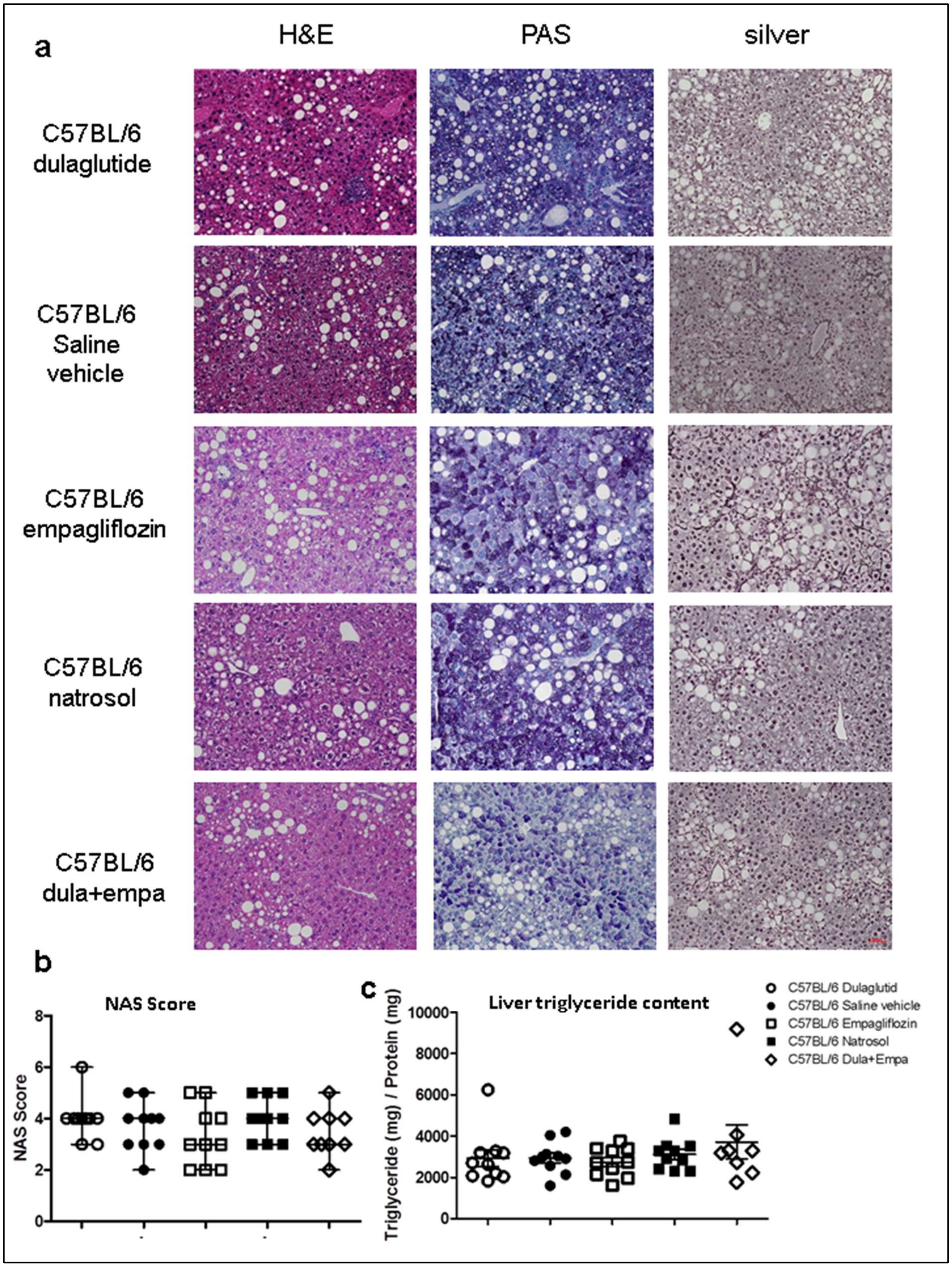

Figure S1: Histological outcome of NAFLD after HFHC diet

(a) Representative images of liver sections stained with haematoxylin and eosin (H&E) for assessment of liver histology, with periodic-acid Schiff reaction (PAS) for assessment of glycogen accumulation and with silver for evaluation of fibrosis (magnification x 20). (b) Histological grading of NASH using the NAFLD-activity score (NAS). Data are presented as median with range. Non-parametric Mann-Whitney test was used for comparison of two groups.

(c) Liver triglyceride content. Data are presented as mean ± SEM. Unpaired t-test with Welsh-correction was used for comparison between of two groups.

C57BL/6 Dulaglutide :n=10, C57BL/6 Saline vehiclel :n=10, C57BL/6 Empagliflozin: n=10, C57BL/6 Natrosol:n=10; C57BL/6 Dula+Empa:n=9

Supplementary Figure S2

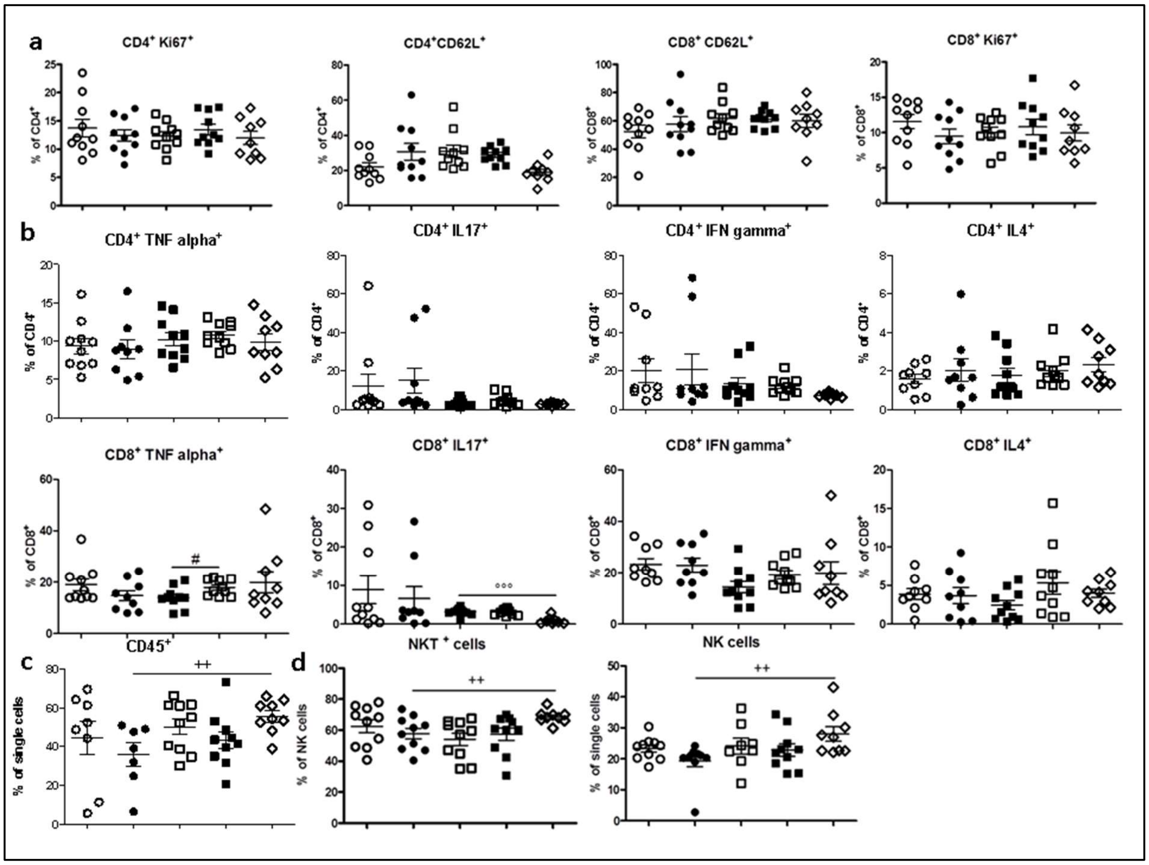

Figure S2: Innate and adaptive immune system

(a) Staining fo $CD4^+$ and $CD8^+$ lymphocytes for Ki67 and CD63

(b) Intercellular staining of intrahepatic lymphocytes determined the content of IFN-gamma+, IL4-+, TNF-alpha and IL17+ subsets of $CD4^+$ and $CD8^+$ T cells.

(c) Staining for $CD45^+$ cells to further analyse the subset of macrophages.

Data are presented as mean ± SEM **(d)** the intrahepatic the intrahepatic subsets of NK-cells.

C57BL/6 Dulaglutide:n=10, C57BL/6 Saline vehicle :n=10, C57BL/6 Empagliflozine: n=10, C57BL/6 Natrosol:n=10; C57BL/6 Dula+Empa:n=9

* *p*< 0.05; ** *p* <0.01; *** *p* <0.001 was used for comparison of Dulaglutide vs. Saline vehicle

$^{\#}$ *p*< 0.05; $^{\#\#}$ *p* <0.01; $^{\#\#\#}$ *p* <0.001 was used for comparison of Empagliflozin vs Natrosol

$^{+}$ *p*< 0.05;$^{++}$ *p* <0.01; $^{+++}$ *p* <0.001 was used for comparison of Dula+Empa vs Saline vehicle

$^{\circ}$ *p*< 0.05;$^{\circ\circ}$ *p* <0.01; $^{\circ\circ\circ}$ *p* <0.001 was used for the comparison of Dula+Empa vs. Empagliflozin

Supplementary Figure S3

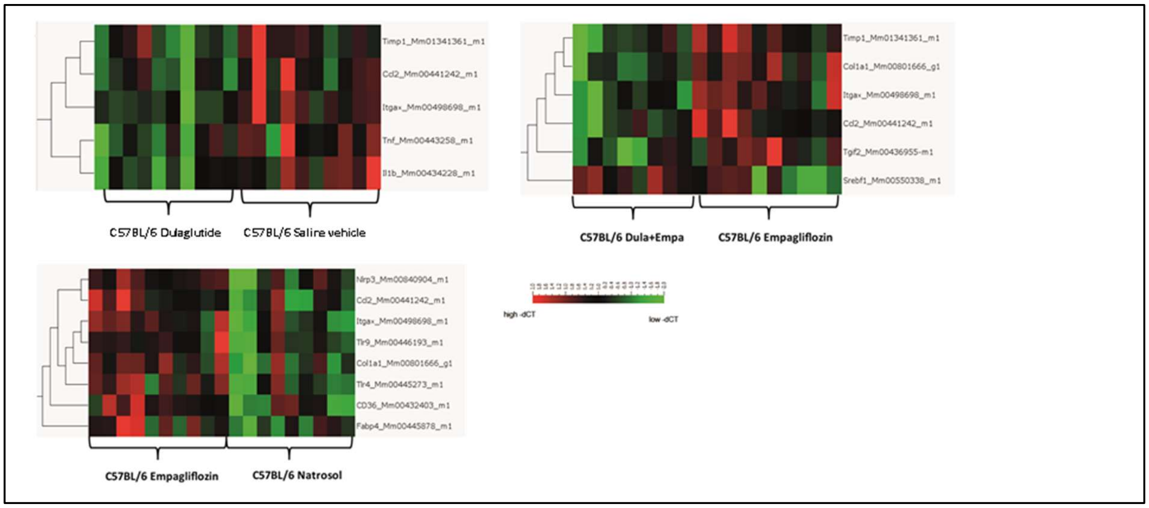

Figure S3: Heat map

Heat map and PCA analysis of the –delta Ct values of genes ($p<0.05$ and $q<0.2$).

Supplementary Figure S4

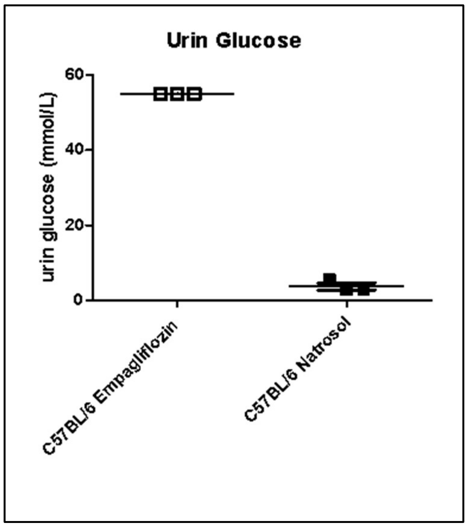

Figure S4: Glucosuria after empagliflozin treatment

To prove the biological activity of empagliflozin, we tested the glucosuria using Combur[9] test. Here, we could show increased glucosuria after empagliflozin gavage compared to natrosol gavage, which proves the biological activity of empagliflozin.

Supplementary Table S1

**table 1**	C57BL/6 Dulaglutide	C57BL/6 Saline vehicle	C57BL/6 Empagliflozin	C57BL/6 Natrosol	C57BL/6 Dula+Empa
Ballooning	1	1	1	1	1
Lobular inflammation	1	1	1	1	1
Steatosis	2	2	1	1	2
NAS Score	4	4	3	4	3
Fibrosis	0	0	1	1	1

Table S1: Histological Scoring for NASH
Details of the histological scoring for NASH for ballooning, lobular inflammation, steatosis and fibrosis. Medians are presented for the detailed NAS score and for fibrosis scoring.

Supplementary Table S2

Genera	C57BL/6 Dulaglutide	C57BL/6 Saline vehicle	C57BL/6 Empagliflozin	C57BL/6 Natrosol	C57BL/6 Dula+Empa	significance
Acinetobacter	0,02	0,00	0,00	0,00	0,00	^
Aerococcus	0,37	0,00	0,00	0,04	0,10	°°° *** #
Akkermansia	1,31	0,10	0,55	0,41	0,29	*** ^
Alistipes	0,39	0,05	0,44	0,29	0,89	*
Allobaculum	49,20	79,15	67,67	69,33	58,72	
Anaerotruncus	0,04	0,01	0,01	0,02	0,06	
Anaerovorax	0,04	0,01	0,01	0,02	0,02	
Bacteroides	3,86	0,31	3,41	2,14	3,32	*** #
Barnesiella	3,91	0,08	0,76	0,56	3,11	°° ***
Bifidobacterium	0,42	0,10	0,34	0,17	0,65	##
Blautia	0,02	0,03	0,00	0,05	0,01	* #
Burkholderia	0,12	0,00	0,00	0,00	0,00	
Butyricicoccus	0,01	0,00	0,01	0,02	0,01	
Cellulosilyticum	0,00	0,00	0,00	0,00	0,00	
Clostridium IV	0,10	0,02	0,06	0,07	0,09	
Clostridium sensu stricto	2,90	0,49	0,35	0,35	2,48	°° *
Clostridium XI	0,00	0,00	0,31	0,98	0,00	°°° *
Clostridium XlVa	0,32	0,26	0,22	0,23	0,27	
Clostridium XlVb	0,42	0,07	0,14	0,27	0,40	°° ***
Clostridium XVIII	4,66	5,85	4,87	5,17	2,24	
Coprobacillus	0,01	0,27	0,16	0,25	0,00	°°° ***
Corynebacterium	0,00	0,00	0,00	0,01	0,20	°°° ^^^
Cupriavidus	0,03	0,00	0,00	0,00	0,00	
Enterococcus	0,13	0,02	0,00	0,01	0,12	°°° **
Enterorhabdus	0,04	0,02	0,02	0,05	0,01	
Erysipelotrichaceae_ incertae_sedis	0,00	0,00	0,00	0,01	0,00	
Escherichia/Shigella	0,25	0,01	0,00	0,01	0,08	°°° **
Facklamia	0,00	0,00	0,00	0,00	0,01	
Flavonifractor	0,06	0,01	0,06	0,07	0,11	^
Gemella	0,00	0,00	0,00	0,01	0,00	#
Holdemania	0,03	0,01	0,02	0,03	0,01	
Isobaculum	0,19	0,06	0,02	0,01	0,08	°°°
Lachnospiracea_ incertae_sedis	0,06	0,00	0,00	0,00	0,00	*** ^^^

Genera	C57BL/6 Dulaglutide	C57BL/6 Saline vehicle	C57BL/6 Empagliflozin	C57BL/6 Natrosol	C57BL/6 Dula+Empa	significance
Lactobacillus	4,97	3,78	5,86	3,08	6,42	#
Macrococcus	0,00	0,01	0,00	0,04	0,00	** ##
Marvinbryantia	0,36	0,06	0,05	0,16	0,11	
Methylobacterium	0,00	0,00	0,00	0,00	0,01	°° ^^^
Olsenella	0,59	0,66	0,00	0,00	0,01	^^^
Oscillibacter	1,03	0,02	0,40	0,36	0,99	***
Parabacteroides	0,86	0,07	0,91	0,73	1,58	***
Parasutterella	0,84	0,06	0,50	0,58	0,65	***
Pseudoflavonifractor	0,12	0,01	0,06	0,02	0,19	° **
Roseburia	0,01	0,04	0,00	0,00	0,00	**
Ruminococcus	0,15	0,00	0,00	0,00	0,00	*** ^^
Staphylococcus	0,15	0,02	0,19	0,83	0,42	^
Streptococcus	0,00	0,01	0,01	0,05	0,00	° *
Streptophyta	0,42	0,00	0,00	0,07	0,00	
unclassified Bacteria	0,33	0,22	0,19	0,23	0,26	
unclassified Bacteroidales	1,91	0,00	1,52	1,06	0,01	°°° *** ^^^
unclassified Bacteroidetes	0,03	0,00	0,01	0,51	0,75	°°° ^^^ ###
unclassified Clostridia	0,00	0,00	0,00	0,00	0,00	
unclassified Clostridiaceae 1	0,00	0,00	0,00	0,00	0,00	
unclassified Clostridiales	7,34	2,56	4,20	4,70	4,51	
unclassified Clostridiales_Incertae Sedis XIII	0,00	0,00	0,00	0,00	0,00	
unclassified Coriobacteriaceae	0,73	0,59	0,61	0,61	0,46	
unclassified Enterobacteriaceae	0,08	0,00	0,01	0,04	0,28	°°° ** +
unclassified Erysipelotrichaceae	0,05	0,07	0,09	0,05	0,03	°°°
unclassified Firmicutes	1,18	1,84	1,13	0,88	0,82	*** #
unclassified Lachnospiraceae	5,77	1,85	2,80	2,04	5,28	#
unclassified Lactobacillaceae	0,00	0,00	0,00	0,00	0,00	
unclassified Lactobacillales	0,00	0,00	0,00	0,00	0,00	
unclassified Peptococcaceae 1	0,40	0,23	0,35	0,47	0,22	
unclassified Porphyromonadaceae	0,01	0,00	0,00	0,00	0,01	
unclassified Prevotellaceae	0,00	0,00	0,00	0,01	0,00	
unclassified Rhodocyclaceae	0,00	0,00	0,00	0,01	0,00	
unclassified Ruminococcaceae	3,78	0,97	1,64	2,88	3,71	*

Table S2: Analyzing the relative abundance of different genera.

Detailed analyses of the relative abundance of different genera for each treatment group. Unpaired t-test with Welsh-correction was used for comparison between treatment and control group, one-way ANOVA with Tukey´s multi comparison test was used for differences between treatment groups.

* *p*< 0.05; ** *p* <0.01; *** *p* <0.001 was used for comparison of Dulaglutide vs. Saline vehicle.
$^{\#}$ *p*< 0.05; $^{\#\#}$ *p* <0.01; $^{\#\#\#}$ *p* <0.001 was used for comparison of Empagliflozin vs Natrosol
$^{+}$ *p*< 0.05; $^{++}$ *p* <0.01; $^{+++}$ *p* <0.001 was used for comparison of Dula+Empa vs Dulaglutide
$^{\circ}$ *p*< 0.05; $^{\circ\circ}$ *p* <0.01; $^{\circ\circ\circ}$ *p* <0.001 was used for the comparison of Dula+Empa vs. Empagliflozin
